# Analysis of circulating cell-free DNA identifies *KRAS* copy number gain and mutation as a novel prognostic marker in Pancreatic cancer

**DOI:** 10.1038/s41598-019-47489-7

**Published:** 2019-08-12

**Authors:** Sumitra Mohan, Mahmood Ayub, Dominic G. Rothwell, Sakshi Gulati, Bedirhan Kilerci, Antoine Hollebecque, Hui Sun Leong, Nigel K. Smith, Sudhakar Sahoo, Tine Descamps, Cong Zhou, Richard A. Hubner, Mairéad G. McNamara, Angela Lamarca, Juan W. Valle, Caroline Dive, Ged Brady

**Affiliations:** 1 Clinical Experimental Pharmacology Group, Cancer Research UK Manchester Institute, University of Manchester, Alderley Park, SK10 4TG Macclesfield, UK; 2 Computational Biology Support, Cancer Research UK Manchester Institute, University of Manchester, Alderley Park, M20 4BX Macclesfield, UK; 3 Medical Oncology Department, The Christie NHS Foundation Trust; Division of Cancer Sciences, University of Manchester, M20 4BX Manchester, United Kingdom; 4 Division of Cancer Sciences, University of Manchester, M20 4BX Manchester, UK

**Keywords:** Tumour biomarkers, Cancer genomics

## Abstract

Serial biopsy of pancreatic ductal adenocarcinoma (PDAC), to chart tumour evolution presents a significant challenge. We examined the utility of circulating free DNA (cfDNA) as a minimally invasive approach across a cohort of 55 treatment-naïve patients with PDAC; 31 with metastatic and 24 with locally advanced disease. Somatic mutations in cfDNA were detected using next generation sequencing in 15/24 (62.5%) and 27/31 (87%) of patients with locally advanced and metastatic disease, respectively. Copy number changes were detected in cfDNA of 10 patients of whom 7 exhibited gain of chromosome 12p harbouring *KRAS* as well as a canonical *KRAS* codon 12 mutation. In multivariable Cox Regression analysis, we show for the first time that patients with *KRAS* copy number gain and *KRAS* mutation have significantly worse outcomes, suggesting that this may be linked to PDAC progression. The simple cfDNA assay we describe will enable determination of the presence of *KRAS* copy number gain and *KRAS* mutations in larger studies and clinical trials.

## Introduction

**Figure 1:**
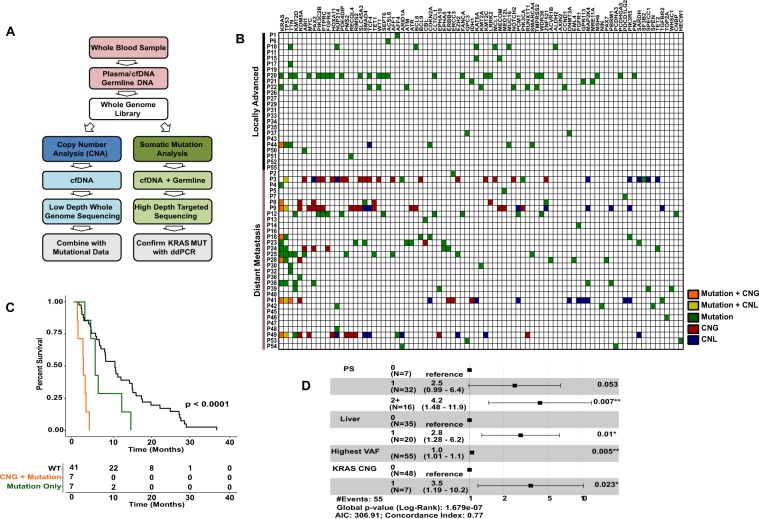
(**A**) Sample Workflow. This flowchart explains the workflow used in this study starting from whole blood samples collected in this study to the analysis performed. (**B**) Combined copy number and mutational analysis of cfDNA. Combined mutational and copy number plot for the 85 genes that were positive for at least 1 mutation across all 55 cfDNA samples analysed. Boxes coloured orange represent mutation and a copy number gain (CNG), yellow mutation and copy number loss (CNL), green mutation, red CNG, and blue for CNL. (**C**) Kaplan-Meier analysis of the overall survival according to *KRAS* mutation alone (7/55), *KRAS* mutation and copy number gain (7/55), along with *KRAS* wild-type (41/55) in 55 patients revealed best prognosis for patients with *KRAS* wild-type, with a median survival of 10.6 months, followed by patients with *KRAS* mutation alone with a median survival of 5.6 months. Patients with both the *KRAS* mutation and copy number gain had the worst prognosis with a median survival of 2.5 months (overall Log-rank test p-value = < 0.0001). *KRAS* WT vs *KRAS* MUT only Log-rank p-value = 0.0610, *KRAS* WT vs *KRAS* CN gain + MUT Log-rank p = value = 0.0012 and *KRAS* MUT only vs *KRAS* CN gain + MUT Log-rank p-value = < 0.0001. (**D**) Hazard ratios for the factors that were used in a multivariable analysis.

Pancreatic ductal adenocarcinoma (PDAC) is an aggressive disease with <7% 5-year survival^1^ and increasing worldwide incidence^2^. Poor patient outcomes are attributed to several factors, including late diagnosis, chemotherapy resistance and the absence of druggable targets to improve patient outcomes^3^. Obtaining tumour biopsies is challenging and carbohydrate antigen 19-9 (CA 19-9), the only approved circulating biomarker for routine clinical management of PDAC (National Comprehensive Cancer Network [NCCN] guidelines) is limited by sub-optimal sensitivity and specificity. More recently, circulating cell free DNA (cfDNA) has been proposed as a minimally invasive alternative to traditional blood-based protein biomarkers and invasive tissue biomarkers for many solid cancer types, including PDAC^4,5^. A previous study detected *KRAS* mutations in cfDNA of 58.9% of patients with PDAC with distant metastasis and 18.2% of patients with locally advanced disease^6^. In this pilot study, we evaluated targeted *KRAS* sequencing and broad next-generation sequencing (NGS) analysis of 641 cancer-associated genes in the cfDNA of 55 patients with PDAC to evaluate the potential clinical utility of cfDNA in PDAC (Fig. 1A).

## Results

A total of 55 treatment-naïve patients with PDAC were identified (between Feb 2011 to Apr 2014); 24 with locally advanced disease and 31 with metastatic disease. The clinical details including age, gender, performance status and metastatic sites are provided in the Supplementary Table 1.

**Figure 2:**
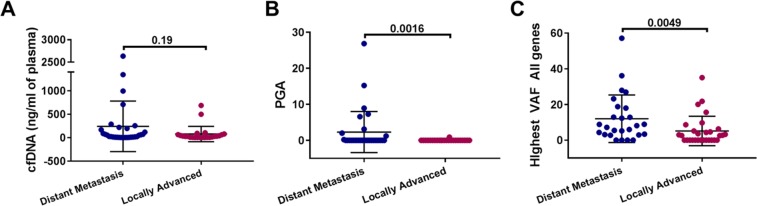
Comparison between patients with distant metastasis and locally advanced disease for (**A**) Yield of cf DNA in ng/ml of plasma (**B**). Percent Genome Amplified (PGA) and (**C**) highest VAF.

No somatic mutations or copy number alterations were detected in 16 non-cancer controls (Table 1). No significant differences were observed in yield of cfDNA detected between the 31 patients with metastatic and 24 with locally advanced PDAC (p-value = 0.19; Fig. 2). From cfDNA NGS, both CNA and somatic mutations were elevated in the patients with metastatic disease compared to the patients with locally advanced disease (p-values of 0.0164 and 0.0049, respectively, Fig. 2B,C). Somatic mutations were detected in 87% (27/31) and 54% (13/24) of the samples from patients with metastatic and locally advanced disease, respectively. Known non-synonymous activating *KRAS* mutations, confirmed by ddPCR, were detected in 35% (11/31) and 12.5% (3/24) of samples from patients with metastatic and locally advanced disease respectively. In addition to the 14 mutations detected by NGS, a further seven *KRAS* mutations (four metastatic, three locally advanced) were detected using ddPCR, which were below the 2.5% VAF (Variant Allele frequency) threshold used for NGS analysis (Fig. 1B). In keeping with previous studies, NGS of cfDNA from the patiens with metastatic disease also identified canonical *TP53* and *KMT2D* mutations at frequencies of 29% (9/31) and 16% (5/31) respectively^6^ (Fig. 1B).

**Table 1:** Univariate and multivariable Cox regression analysis for prediction of OS.

Parameter		At risk group	HR [95% CI]	p-value
**Univariate analysis**				
Gender		Male (vs Female)	1.68 [0.97, 2.89]	0.064
Age		Continuous	0.99 [0.97, 1.02]	0.545
ECOG Performance status		1 (vs 0)	1.77 [0.77, 4.08]	0.179
		>=2 (vs))	4.34 [1.72, 10.96]	0.002
ORR		Response (vs no response)	0.31 [0.13, 0.76]	0.010
Metastasis	Number of metastatic sites	>=2 sites (vs <2 sites)	1.31 [0.66, 2.62]	0.44
	Liver metastasis	Yes (vs No)	3.05 [1.70, 5.51]	<0.001
	Lung metastasis	Yes (vs No)	0.77 [0.35, 1.67]	0.505
	Other sites	Yes (vs No)	1.61 [0.68, 3.83]	0.283
WCC^$^		Continuous	1.18 [1.11, 1.25]	<0.001
Neutrophils^$^		Continuous	1.19 [1.11, 1.26]	<0.001
Lymphocytes		Continuous	0.72 [0.46, 1.12]	0.143
LDH		Continuous	1.001 [1.000, 1.003]	0.003
Ca 19.9 (Log2)		Continuous	1.07 [0.99, 1.16]	0.091
CNA		Continuous	7.09 [3.19, 15.78]	<0.001
Mutational burden	Number of mutations^$^	Continuous	1.06 [1.01, 1.12]	0.028
	Number of KRAS mutations^$^	Continuous	1.11 [1.07, 1.16]	<0.001
	KRAS mutations present	Yes (vs No)	3.46 [1.76, 6.77]	<0.001
	KRAS copy number gain	Yes (vs No)	10.94 [3.85, 31.08]	<0.001
	Highest VAF	Continuous	1.07 [1.04, 1.10]	<0.001
**Multivariable analysis**				
ECOG Performance status		1 (vs 0)	2.51 [0.98, 6.38]	0.053
		>=2 (vs 0)	4.20 [1.48, 11.94]	0.007
Metastasis	Liver metastasis	Yes (vs No)	2.83 [1.28, 6.24]	0.010
Mutational burden	KRAS copy number gain	Yes (vs No)	3.47 [1.19, 10.17]	0.023
	Highest VAF	Continuous	1.05 [1.01, 1.08]	0.005

Abbreviations: ORR, objective response rate (clinical outcome variable); VAF: variant allele frequency; ECOG, Eastern Cooperative Oncology Group; WCC: white cell count; $, Excluded from stepwise model building due to collinearity.

Measurable copy number alterations (CNA) were detected in 10 of the 55 patients’ cfDNA samples (nine metastatic, one locally advanced), of whom seven exhibited a gain in chromosome 12p that harbours *KRAS* (Fig. 1B). All seven PDAC cfDNA samples with copy number gain (CNG) of *KRAS* also exhibited non-synonymous somatic mutations in *KRAS* (Fig. 1B and Supplementary Table 1).

Kaplan-Meier analysis of overall survival (OS) based on *KRAS* mutation alone (7/55), *KRAS* mutation and CNG (7/55) and with *KRAS* wild-type (34/55), revealed best prognosis for patients with *KRAS* wild-type (median survival 10.6 months), followed by patients with *KRAS* mutation without CNG (median survival 5.5 months). The worst prognosis was associated with the combination of a *KRAS* mutation and CNG (median survival 2.5 months, Log-rank p-value < 0.0001; Fig. 1C,D). Univariate analysis identified highest VAF (any gene), *KRAS* CNG, performance status (PS) and presence of liver metastases as significant factors for shorter survival with a p-value < 0.05. Stepwise multivariable analysis (Table 1) identified *KRAS* CNG and mutation as an independent predictor for shorter survival.

## Discussion

In this pilot study of 55 patients with PDAC, we applied NGS and ddPCR to cfDNA to establish which readouts, if any, are linked to clinical outcomes. Although we see a relatively short median survival of 7.99 months compared to the 19.77 months reported in a TCGA study^7^, this most likely reflects differences in staging with the TCGA cohort comprising operable localised disease whereas our cohort includes patients with locally advanced and metastatic disease, resulting in a shorter median survival, in line with those reported by other groups^8,9^. Analysis of cfDNA from each patient revealed the presence of a canonical *KRAS* somatic mutation, which was determined by ddPCR and was found to be 38% (21/55) overall; 48% (15/31) in metastatic disease and 25% (6/24) in locally advanced disease, in keeping with other published studies^6^. Although our detection rate of 38% for the presence of a *KRAS* mutation in patient cfDNA is in line with other reports, there is considerable variation in reported frequencies (27~93%)^10,11^ which may reflect the methodologies employed, as well as the variability of *KRAS* allelic ratios in the tumour^10^ and the low ctDNA burden associated with pancreatic cancer^12^. Analysis of a larger cohort with a consistent specified cfDNA methodology is required to assess the affect of *KRAS* variation on the accuracy of prognosis.

As expected, from the threshold of detection used for the targeted NGS in this study (2.5%)^13^, only 14/21 ddPCR positive samples were found to harbour targeted NGS somatic *KRAS* mutations (Supplementary Table 1). However, by extending the NGS analysis to an additional 640 genes, somatic mutations were detected in 71% (39/55) in all samples; 84% (26/31) in metastatic disease and 50% (12/24) in locally advanced disease. The most striking novel observation that emerged from this study was that >10% of patients with PDAC harboured both a *KRAS* mutation and a *KRAS* CNG, and that the latter correlated with a worsened prognosis. Although amplified mutated KRAS has been reported in non-small cell lung cancer (NSCLC) and is also associated with poor clinical outcome^14^, this is the first report in PDAC. In addition to identifying CNG of *KRAS*, we also noted four cases where *TP53* mutations were accompanied by copy number loss (CNL), suggesting that further analysis of a larger patient group may also identify CNL as a prognostic biomarker.

We now have the opportunity to verify these initial results by examining additional patient cfDNAs from the on-going Precision-Panc clinical trial, and serial measurements may inform response to treatment^15^.

Our results demonstrate cfDNA analysis can be used in advanced disease to identify patients with worse prognosis who may benefit from more aggressive chemotherapy. In addition, the identification of *KRAS* CNG and mutation as a poor prognostic factor, could also help to identify patients with resectable disease with higher risk of early tumour relapse, who may benefit from additional staging imaging before surgery (i.e. Magnetic resonance imaging of the liver or 18 fluorodeoxyglucose (FDG)-positron emission tomograph) or potential neo-adjuvant.

## Materials and Methods

### Non-Cancer volunteer and patient blood sample collection

Patients diagnosed with advanced treatment-naïve PDAC were prospectively recruited. Baseline blood samples (before treatment initiation) were collected in Cell-Free™ DNA BCTs (Streck, Omaha, NE), or BD Vacutainer® K_2_EDTA tubes, following receipt of informed consent in compliance with the Declaration of Helsinki and Good Clinical Practice under ethics approval number 07/H1014/96 (approved by Internal Review and Ethics Board of the Manchester Cancer Research Centre BioBank).

### Circulating cell free DNA preparation

Plasma and cfDNA were isolated as previously described^16^. Germline DNA was isolated from EDTA whole blood, using QIAmp Blood Mini Kit (Qiagen, Hilden, Germany) as per manufacturer’s instructions.

### NGS library preparation and sequencing

Whole genome sequencing (WGS) of cfDNA and corresponding germline DNA from the patients as well as non-cancer controls were carried out using the Accel-NGS® 2 S Plus DNA Library Kit as previously described^16^.

### Targeted NGS analysis

Targeted NGS of 641 cancer-associated genes was carried out using Agilent SureSelectXT as described previously^13^.

### Somatic mutation detection from targeted re-sequencing data

Three mutation callers were used: MuTect (version 1.1.5); VarScan (version 2.3.9) and Biomedical Genomics Workbench 4.1 (CLC Bio, Qiagen). Single nucleotide variant (SNV) calls were accepted, if identified by both MuTect and Biomedical Genomics Workbench and indels accepted if identified by both VarScan and Biomedical Genomics Workbench (Fig. 1B). HMMcopy (version 1.8.0) was used to call regions as gained or lost from WGS^16^.

### Droplet digital PCR

Droplet digital PCR (ddPCR) was carried out using a QX200 ddPCR system (Bio-Rad) with ddPCR^TM^ KRAS Screening multiplex kit^17^.

### Statistical analyses

Mann-Whitney t-tests were used to compare cfDNA metrics (cfDNA in ng/ml of plasma, Percent genome amplified [PGA] and Highest VAF) between patients with locally advanced disease and patients with distant metastases. Factors associated with mutational burden and standard clinical and biochemical factors were subjected to Kaplan-Meier survival analysis and univariate Cox proportional hazards regression to predict overall survival (OS), considering the proportionality and linearity assumptions. OS was defined as the time in months between date of first diagnosis of malignancy and time of death. Univariately significant parameters (5% significance level) were included in a multivariable Cox regression analysis (bidirectional stepwise selection based on Akaike information criterion; exclusion of collinear parameters and clinical outcome variable). Statistical analysis was performed using the computing environment R (R Development Core Team, 2005).

### Ethics approval and consent to participate

Blood samples were collected from patients with PDAC following receipt of informed consent in compliance with the Declaration of Helsinki and Good Clinical Practice under ethics 07/H1014/96, after approval from the Internal Review and the Ethics Boards of The Manchester Cancer Research Centre BioBank.

## Supplementary information

Supplementary Information

Table 1

## Data Availability

All the data generated or analysed during this study are included in this published article, or are available from the corresponding author upon reasonable request.
