# Supplementary material for: Analysis of circulating cell-free DNA identifies KRAS copy number gain and mutation as a novel prognostic marker in Pancreatic cancer
Source: Sci Rep. 2019 Aug 12;9:11610. doi: 10.1038/s41598-019-47489-7 (PMC6690979; doi:10.1038/s41598-019-47489-7)
Supplement: Supplementary file 1 — Supplementary Information [file 41598_2019_47489_MOESM1_ESM.pdf]

Analysis of circulating cell-free DNA identifies *KRAS* copy number gain and mutation as a novel prognostic marker in Pancreatic cancer

Sumitra Mohan[1], Mahmood Ayub[1], Dominic G. Rothwell[1], Sakshi Gulati[1], Bedirhan Kilerci[1], Antoine Hollebecque[1], Hui Sun Leong[2], Nigel K. Smith[1], Sudhakar Sahoo[2], Tine Descamps[1], Cong Zhou[1], Richard A. Hubner[3], Mairéad G. McNamara[3,4], Angela Lamarca[3], Juan W. Valle[3,4#], Caroline Dive[1#] and Ged Brady[1#].

[1]Clinical Experimental Pharmacology Group, CRUK Manchester Institute University of Manchester, M20 4BX, UK, [2]Computational Biology Support, CRUK Manchester Institute, University of Manchester, M20 4BX, [3]Medical Oncology Department, The Christie NHS Foundation Trust; Division of Cancer Sciences, University of Manchester, M20 4BX, Manchester, United Kingdom, [4]Division of Cancer Sciences, University of Manchester, M20 4BX, Manchester, UK

# Joint senior authors, correspondence to gerard.brady-2@manchester.ac.uk

Supplementary Information

Supplementary Figure 1: Kaplan-Meier analysis of overall survival according to *KRAS* mutations detected by A) ddPCR assay and B) NGS assay

Supplementary Table1: Summary of patient characteristics

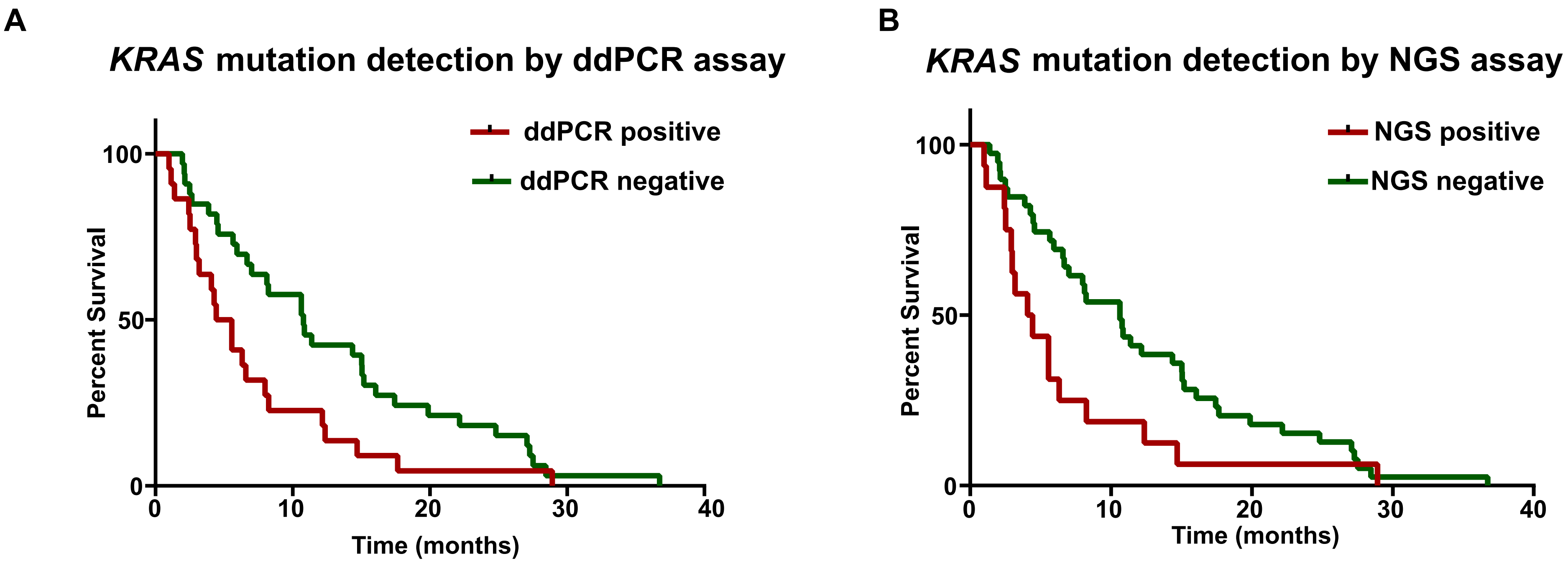

Median OS: 5 months vs 10.7 months
p-value: 0.0222

Median OS: 4.25 months vs 10.625 months
p-value: 0.0363
